# Transforming entomology to adapt to global concerns: 2021 student debates

**DOI:** 10.1093/jisesa/iead064

**Published:** 2023-08-01

**Authors:** Patricia Prade, Ramandeep Kaur Sandhi, Sarah DePaolo Elzay, Katherine Arnold, Victoria Pickens, Andrew Freedman, DeShae Dillard, Sean Gresham, Ashley Morris, Daniela Pezzini, Seun O Oladipupo, Elijah P Carroll, Richard O Murphy, Festus K Ajibefun, Luis M Mendez, Katherine Carroll, Jasleen Kaur, Lillie M Rooney, Kendall Stacey, Yasmin Tavares, Jared E Dyer, Na Xie, Jason Bielski, John Schepis, Kayleigh C Hauri, John J Ternest, Jacob Pecenka, Scott W Gula, Natalie Constancio, Emily Rampone, Mario Luppino, Dowen Jocson, Stephen Onayemi, Emily Rendleman

**Affiliations:** Department of Entomology, Rutgers University, P.E. Marucci Center, Chatsworth, NJ 08019, USA; FMC Corporation, Stine Research Center, 1090 Elkton Road, Newark, DE 19711, USA; Department of Integrative Biology, Oklahoma State University, OK 74078, USA; Department of Entomology Plant Pathology and Weed Science, New Mexico State University, Las Cruces, NM 88003, USA; Department of Entomology, Kansas State University, Manhattan, KS 66506, USA; Department of Entomology and Plant Pathology, North Carolina State University, Raleigh, NC, 27695, USA; Department of Entomology and Plant Pathology, North Carolina State University, Raleigh, NC, 27695, USA; Department of Entomology and Plant Pathology, North Carolina State University, Raleigh, NC, 27695, USA; Entomology and Nematology Department, University of Florida, Gainesville, FL 32611, USA; Department of Entomology and Plant Pathology, North Carolina State University, Raleigh, NC, 27695, USA; Department of Entomology and Plant Pathology, Auburn University, Auburn, AL 36830, USA; Department of Entomology and Plant Pathology, Auburn University, Auburn, AL 36830, USA; Department of Entomology and Plant Pathology, Auburn University, Auburn, AL 36830, USA; Department of Entomology and Plant Pathology, Auburn University, Auburn, AL 36830, USA; Department of Entomology and Plant Pathology, Auburn University, Auburn, AL 36830, USA; Entomology and Nematology Department, University of Florida, Gainesville, FL 32611, USA; Entomology and Nematology Department, University of Florida, Gainesville, FL 32611, USA; Entomology and Nematology Department, University of Florida, Gainesville, FL 32611, USA; Entomology and Nematology Department, University of Florida, Gainesville, FL 32611, USA; Entomology and Nematology Department, University of Florida, Gainesville, FL 32611, USA; Virginia Polytechnic Institute and State University, Blacksburg, VA 24060, USA; Virginia Polytechnic Institute and State University, Blacksburg, VA 24060, USA; Virginia Polytechnic Institute and State University, Blacksburg, VA 24060, USA; Virginia Polytechnic Institute and State University, Blacksburg, VA 24060, USA; Department of Entomology, Michigan State University, East Lansing, MI 48824, USA; Entomology and Nematology Department, University of Florida, Gainesville, FL 32611, USA; Department of Entomology, Purdue University, West Lafayette, IN 47907, USA; Department of Forestry and Natural Resources, Purdue University, West Lafayette, IN 47907, USA; Department of Entomology, Michigan State University, East Lansing, MI 48824, USA; Department of Entomology, Washington State University, Pullman, WA 99164, USA; Department of Entomology, Washington State University, Pullman, WA 99164, USA; Department of Entomology, Washington State University, Pullman, WA 99164, USA; Department of Entomology, Washington State University, Pullman, WA 99164, USA; Department of Entomology, Washington State University, Pullman, WA 99164, USA

**Keywords:** student debates, biological control, insect pest, insect farming

## Abstract

The 2021 Student Debates of the Entomological Society of America (ESA) were held at the Annual Meeting in Denver, CO. The event was organized by the Student Debates Subcommittee (SDS) of the Student Affairs Committee (SAC). The theme of the 2021 Student Debates was “Transforming Entomology to Adapt to Global Concerns”, with 3 topics. Each topic had an unbiased introduction and 2 teams. The debate topics were (i) Nonnative insect introduction is an ethical approach for counteracting proliferation and overpopulation of consumers, (ii) What is the best technology to control undesirable insect pests in urban and agricultural settings? and (iii) Compared to other solutions, like plant-based diets, insect farming is the best method to address rising human global food and nutrient supply demands. Unbiased introduction speakers and teams had approximately 6 months to prepare for their presentations.

## Introduction

During the Entomological Society of America Annual Meeting, the Student Debate Subcommittee (SDS), a subgroup of the Student Affairs Committee (SAC) organizes the student debates. Each year, the SDS chooses 3 debate topics among several topics that are currently being discussed in entomology. The student debates give students the opportunity to showcase their knowledge of the topic selected and cross-examine opposite teams’ ideas. The teams are judged by a panel of 3 judges based on their ability to defend their topics and answer questions from the opposite team, judges, and other meeting attendees. Prior to the debate on each topic, a member of the SDS gives an unbiased introduction (see Parker et al. 2019 for more details on guidelines and rubric). In this article, we provide a summary of the 2021 student debates with unbiased introductions and the teams’ responses to their topics. The student debate topics were:

1) Nonnative insect introduction is an ethical approach for counteracting the proliferation and overpopulation of consumers (pro/con).
2) What is the best technology to control undesirable insect pests in urban and agricultural settings?
3) Compared to other solutions, like plant-based diets, insect farming is the best method to address rising human global food and nutrient supply demands (pro/con).

## Nonnative Insect Introduction Is an Ethical Approach for Counteracting the Proliferation and Overpopulation of Insect Pests

### Unbiased Introduction by Ramandeep Kaur Sandhi and Sarah DePaolo Elzay

Classical biological control (CBC) involves the use of nonnative species as biological control allies and has long been advocated as a pest control method that greatly reduces costs and broad-spectrum pesticide use. Invasive pests cost agricultural and forestry production in the United States up to US$40 billion each year (Pimentel et al. 2005). Most often the natural enemies from the same region as the invasive pests are introduced to parasitize or depredate the invasive pests. A small population of the natural enemy is collected and inoculated into the invaded area for multiplication and semipermanent or permanent pest management. One of the earliest and most successful examples of the use of a biological control agent was the introduction of the Vedalia beetle (*Rodolia cadinalis;* M.) (Coleoptera: Coccinellidae) in California in the 1880s to combat the cottony cushion scale (Sorensen et al. 2019). Additional successful cases have been reported with this type of biological control, but many to only a partial extent (Bellows and Fisher 1999, Hajek and Eilenberg 2018). For example, the use of *Encarsia formosa* (Hymenoptera: Aphelinidae) to control *Trialeurodes vaporariorum* (W.) (Hemiptera: Aleyrodidae) in greenhouses in Europe was highly successful in controlling the devastating whitefly populations (Hoddle et al. 1998).

There are benefits and risks associated with biological control. Nonnative introductions can greatly reduce pesticide use against insect pests, improving environmental safety. Indeed, pesticide application costs the United States approximately US$10 billion each year (Pimentel and Greiner 1997). Pesticides can accumulate in nontarget organisms, water, air, and soil, harming entire ecosystems. For example, chlorantraniliprole, a harmful pesticide contaminant, was found in 91% of milkweed samples visited by the threatened monarch in a recent study in California (Halsch et al. 2020). The use of nonnative predators and parasitoids against invasive pest species can greatly reduce these harmful system-wide effects of pesticide use. Specialist predators and parasitoids are particularly beneficial in controlling invasive pest species. These specialists tend to cause fewer harmful, nontarget effects on native species (Messing and Wright 2006). In a review of cases of biological control, over 80% protected biodiversity within the targeted ecosystem and 62% of those completely controlled the targeted invasive pest (van Driesche et al. 2010). Biological control uses nonnative parasitoids and predators is highly successful, affordable, and decreases the negative impacts of pesticide use across ecosystems.

Negative impacts of biological control on nontarget species are well documented. This approach can take considerable time, making it slower than pesticides for the effective suppression of a pest. Biological control agent rearing and releasing can be expensive and require continuous augmentative release when the agent is not intended to or does not establish a natural breeding colony (Collier and Van Steenwyk 2004). Additionally, although minimal nontarget effects have been reported, there are some chances of nontarget effects due to these strategies. For example, the Asian ladybird beetle, *Harmonia axyridis* (P.) (Coleoptera: Coccinellidae), introduced to control aphid pests, has caused declines among native ladybird beetles across many regions of the United States (Koch and Galvan 2007). Many nonnative biological control agents have been generalist predators and parasitoids, which have devastated native, nontarget species (Howarth 1991). The negative impacts of nonnatives on biodiversity have created a shift in public opinion regarding biological control. However, some researchers emphasize that nontarget impacts are rare when specialist predators and parasitoids are used and that continued monitoring is essential to confirm nontarget effects remain controlled (Messing and Wright 2006). This debate will address the pros and cons associated with the introduction of nonnative species as a mechanism of biological control.

## Team 1 Stance: Nonnative Insect Introduction Is an Ethical Approach for Counteracting the Proliferation and Overpopulation of Insect PestsTeam Members: Andrew Freedman, DeShae Dillard, Sean Gresham, Ashley Morris, Daniela PezziniFaculty Advisor: Dr. Hannah Burrack, North Carolina State University

The introduction of nonnative insects to counteract the proliferation of consumers is an important Integrated Pest Management (IPM) tool in mitigating the negative impacts of invasive herbivores on agricultural and natural systems. This introduction, termed classical biological control can be ethically implemented if (i) nonintervention constitutes more risk than intervention, (ii) alternative tactics are ineffective or present greater risk, (iii) conflicts of interests between stakeholders are addressed, and (iv) stakeholders are meaningfully engaged through transparent communication, education, and needs recognition (Lockwood 1996, Bale et al. 2008). Our intention is to act ethically towards our values of human well-being and food security under these guidelines and we recognize CBC is one important tool that can be integrated with and enhance alternative control measures.

Over the past 120 yr over 5,000 CBC introductions have been made throughout the world (Bale et al. 2008), many of which have successfully established and suppressed pest populations (Márquez et al. 2019, Andersen et al. 2021), with very limited reports of negative impacts (Bale et al. 2008, Hajek et al. 2016). Exotic biological control agents remain important to sustainable agricultural practices throughout the world, especially in Africa, neotropics, and island nations (Cock et al 2009, Colmenarez et al. 2018). One major driver for CBC is the predominance of invasive pest species resulting from intentional and unintentional introductions of exotic species (Bradshaw et al. 2016, Valentin et al. 2017). The global cost of invasive insects is estimated US$70 billion annually and may be greater due to a raft of unknown interactions (Bradshaw et al. 2016). Hence some of the greatest successes in CBC implementation have been in island ecosystems such as Galapagos (Hoddle et al. 2013), neotropical region (Colmenarez et al. 2018), or the Pacific (Wyckhuys et al. 2020) which are at greatest risk from invasive pests.

All forms of intervention carry inherent risk and the potential to modify natural ecosystems (Kaufman and Wright 2017), including CBC (Hajek et al. 2016). For example, neonicotinoids were readily adopted because they have low mammalian toxicity when compared to other insecticides (i.e., organophosphates), but widespread use has caused deleterious effects across different groups of insects (Main et al. 2018). Robust risk assessment of CBC is therefore necessary: it should be ecologically based, utilize cost-benefit analysis, and incorporate public advocacy (Warner 2016). The downside risks need to be evaluated against the costs of nonintervention and alternative management strategies (Delfosse 2005). A few risk assessment tools are available and are continually improving (Kimberling 2004, Barratt et al. 2010, Kaufman and Wright 2017), and these assessments should inform stakeholders of the following: (i) host specificity, (ii) susceptibility of the agent to mitigation, (iii) extensive long-term monitoring, and (iv) necessity in restoring well-being of native and integrated ecosystems (Lockwood 1996).

Public advocacy empowers stakeholders by ensuring the conflicts of interest are ascertained before intervention to gauge support outside of the scientific community (Salom et al. 2019). Effective public advocacy and involvement has been implemented by New Zealand where land managers petition for control, concerned citizens have direct input, and scientists function to assess risk for decision-making (Warner 2016). The ethical implementation of any approach is determined by the process in which the decisions are made, and not simply a measure of successful outcomes (Johnson 1996).

Although CBC is not the solution to all pest issues, we believe it is an effective tool that can be implemented ethically. When implementing CBC, or any control tactic, decision-making authorities should take into consideration risks to stakeholders and follow ethical frameworks that quantify risk while prioritizing human well-being and food security.

## Team 2 Stance: Nonnative Insect Introduction Is Not an Ethical Approach for Counteracting the Proliferation and Overpopulation of Insect PestsTeam Members: Seun O. Oladipupo, Elijah P. Carroll, Richard O. Murphy, Festus K. Ajibefun, Luis M. MendezFaculty Advisor: Dr. David W. Held, Auburn University

Classical biological control is the importation and release of an exotic species outside of its natural range for the purpose of suppressing pest populations (Howarth 1991). Classical biological control became internationally popular as a cheap alternative to insecticides in the early 1900s when pests were controlled with low costs (Simberloff 2012). For example, significant reductions of cottony cushion scale in orange groves resulted from the release of the vedalia ladybeetle, *Novius cardinalis* (Mulsant) (Coleoptera: Coccinellidae), in California (Howarth 1991), providing an example of how expensive pest problems may be solved through inexpensive methods.

However, since 1980s growing concerns over unpredictable consequences have increased skepticism about CBC as a management tactic. These concerns include the unpredictability of (i) host specificity and its relation to nontarget impacts to natives, (ii) reversibility of established agents, and (iii) their dispersal into novel areas (Simberloff 2012). Furthermore, preliminary testing procedures of potential agents rely heavily on host specificity testing alone, which is not sufficient to predict potential impacts (Taylor and Snyder 2020), and a failure to conduct postrelease evaluations have resulted in significant negative impacts on the environment and public health to be overlooked (De Clercq 2002). Lastly, based on metadata, successes of biological control are low (Cock et al. 2016), ultimately placing the stakeholder at risk of monetary expenses resulting from yield losses and failed management practices.

The general assumption has been that the risk of potential ecological impact increases with polyphagy of the biocontrol agent. Choice-assays are expensive, may not provide ecologically significant data, and are limited in scope by testing direct effects on a few nontarget species (De Clercq 2002). A review based on 9 species of exotic natural enemies, ranging in degrees of host specificity, suggests that when both direct and indirect effects are accounted for, there is no trend between host specificity and ecological safety (Taylor and Snyder 2020). Indirect effects via apparent competition are observed more for specialists relative to generalists and have greater impacts on the species in which they interact with via spillover (Taylor and Snyder 2020). Nontarget impacts become more unpredictable as species disperse into new environments and evolve over time (Simberloff and Stiling 1996a, 1996b). The only predictable outcome to biocontrol releases is once biocontrol agents are established, their residence is irreversible which could increase chances for nontarget effects (Howarth 1991).

Broadly speaking, there are 29 proposed hypotheses regarding the mechanisms leading to establishment of a nonnative species (Shulz et al. 2019). Like nontarget impacts, they are hard to accurately predict. This unpredictability can be observed by low percentages of success in species establishment, and even lower percentages in satisfactory control of target pest populations (Kenis et al. 2017). This unpredictability of biocontrol agents in successfully controlling a pest population may have negative economic impacts on stakeholders, however, no study has evaluated the economic impact of failures in this system. Negative impacts on public health have been observed for *Harmonica axyridis* Pallas (Coleoptera: Coccinellidae), for example, via structural infestations and consequential discomfort and health hazards to homeowners (Huelsman et al. 2002). Limited data has been accumulated through a survey of Ohio state residents and no economic value has been obtained for this problem (Huelsman et al. 2002).

Delfosse (2005) argues consequentialist ethics should be used for biological control, where all the reasonable alternatives are listed, and future consequences are compared. The current dilemma in ethical biological control is the unpredictability of postrelease outcomes, including nontarget effects and the likelihood of successful management. Therefore, it is just to rely on historical data, which lends to the conclusion CBC is an intractable system leading to unpredictable and unquantifiable negative impacts.

## What Is the Best Technology to Control Undesirable Insect Pests in Urban and Agricultural Settings?

### Unbiased Introduction by Katherine Arnold

The world population is predicted to reach 9.7 billion people by 2050 (UNDESA 2022), and as the world population increases, food consumption also increases, with a projection of 35–56% increase in food demand by 2050 (Gouel and Guimbard 2019, van Dijk et al. 2021). However, agricultural land has been decreasing (Smith et al. 2010). The US Census shows that farmlands decreased from 914,527,657 in 2012 to 900,217,576 in 2017 (Dempsey 2019). While this decrease in the United States is concerning, we currently have enough land to produce the estimated 9 billion people globally, both in a healthy and sustainable manner (Smith et al. 2010). While this is true, the FAO estimates that around 20% to 40% of the worldwide crop production is lost due to pests (FAO 2019), with US$220 billion of this loss caused by plant diseases, US$ 70 billion due to invasive insects (FAO 2019), and 35% of crops lost is due to preharvest pests (Popp et al. 2013). To provide food for the ever-growing population with reduced farmland, we need to begin to decrease the losses caused by pests. Many practices can be used to control pests and increase food quantity, two of which are chemical control, a conventional well used and known practice, and volatile organic compounds (VOCs) a new and upcoming practice. Both practices have pros and cons, as any part of farming does.

Chemical control of pests is a common practice that has been implemented for decades starting in the 1930s. Widespread use of synthetic chemicals in the US to control pests did not begin until the end of World War II (UC San Diego 2000). Farms began to see crop yield increase due to pest damage decreasing. From 1965 to 1990 the use of synthetic pesticides doubled the theoretical yield of crops harvested (42–70%) (Popp et al. 2013). As of 2020, pesticide trade reached a value of US$41.1 billion (FAO 2022). Pesticides have several benefits, as they help to decrease crop loss, however, the use of pesticides also comes with risks. Pesticides have 3 harmful categories to applicators: acute effect, delayed effect, and allergic effect (UC San Diego 2000). Not only there are risks when applying some pesticides, but we have also begun to see resistance to different modes of action. There are several species of insects and mites that have been documented as having developed resistance to pesticides. The ways to get around pesticide resistance are to apply more pesticides, combine pesticides with different modes of action, increase the frequency of application, or select a more toxic pesticide (UC San Diego 2000).

Volatile organic compounds have begun to emerge as a new alternative to applying synthetic chemicals. These naturally derived chemicals have a less harmful effect to applicators than those of conventional chemistries. Volatile organic compounds also offer a new way to combat pests that have developed resistance to synthetic chemicals. One example is the control of glasshouse whitefly, *Trialeurodes vaporariorum* Westwood (Hemiptera: Aleyrodidae). The glasshouse whitefly has developed resistance to conventional chemicals (Conboy et al. 2020), while VOCs have shown to serve as an effective deterrent for glasshouse whiteflies and increased crop yield up to 32% (Conboy et al. 2020).

There are many different methods of controlling pests, each having positive and negative effects associated with them. The 2 above methods are just 2 of many available options. Chemical control is a common and well-established practice, but VOC is a new and upcoming method of controlling pests. Our goal is to help our farmers increase production in a safe and sustainable manner, as food production must increase using less space.

## Team 3 Stance: The Use of Volatile Organic Compounds Is the Best Technology to Control Undesirable Insect Pests in Urban and Agricultural SettingsTeam members: Katherine Carroll, Jasleen Kaur, Lillie M. Rooney, Kendall Stacey, Yasmin TavaresFaculty Advisor: Dr. Rachel Mallinger, University of Florida

The management of arthropod pests is crucial for human comfort, food security, and health. Insecticides are often the primary choice of both homeowners and crop producers to control pests. However, the development of insecticide resistance in pestiferous insects is a growing issue that requires the development of new technologies and chemical structures to maintain control (Sammataro et al. 2005, Pickett and Khan 2016). Additionally, insecticides can have unintentional effects on nontarget organisms, and can pollute ecosystems (Goulson 2013, Brilli et al. 2019, Humann-Guilleminot et al. 2019). Neonicotinoids, a class of insecticides commonly used due to their low mammal toxicity, have been shown to have lethal effects on pollinators. Even low levels of chronic exposure have been shown to cause long-term behavioral changes and reduced fitness in beneficial insects, such as bumblebees (Wood and Goulson 2017). Pro-insecticides, considered highly selective and environmentally benign because they only gain toxicity after being metabolized, can negatively impact beneficial insects, small birds, and small mammals (Branch 2006, Goulson 2013). Insecticides may also have detrimental effects on aquatic ecosystems and have been shown to negatively impact human health (Goulson 2013, Donley 2019). These nontarget effects, driven by an overreliance on insecticides and the need for reliable control over adaptable arthropod pests, necessitate the development of technologies that will work synergistically with chemical control to manage pests while reducing off-target impacts.

Volatile organic compounds are naturally occurring chemicals produced by plants to combat herbivory, though many have been synthetically formulated. Volatile organic compounds, already an integral component of plant defenses and plant-insect networks, pose less of a threat to ecosystem stability and nontarget organisms when compared to the introduction of foreign insecticides. Preliminary evidence suggests that some forest VOCs, such as limonene and pinene, can even have positive effects on human health and well-being (Antonelli et al. 2020). As the body of research in VOCs grows, so does the potential for their use in new Integrated Pest Management (IPM) technologies. VOCs work in a myriad of ways, from the direct repulsion of herbivores (Kos et al. 2013), to the attraction of natural enemies (Jaworski et al. 2019), elicit induced systemic resistance (ISR) to plant diseases (Song and Ryu 2013), and the initiation of preemptive defenses in surrounding plants (Skoczek et al. 2017). VOCs can be used as a way of monitoring pests (Laothawornkitkul et al. 2008), attracting biocontrol agents, and to attract pests to nonhost crops via intercropping with aromatic plants (Huang et al. 2020). Some popular VOC manipulation techniques include supplementing plant VOCs using synthetic VOCs, applying defense elicitors, like jasmonic acid or salicylic acid, to increase plant VOC emissions, and using VOCs to monitor pests in the field (Himanen et al. 2017). Currently, research is being done on the potential of conventional breeding and genetic engineering to produce plants with greater beneficial VOCs emissions (Kos et al. 2013, Pickett and Khan 2016, Turlings and Erb 2018).

Field and lab studies indicate that certain VOCs may elicit multiple benefits at once, such as both ISR to plant diseases and natural enemy attraction (Song and Ryu 2013). VOCs have the potential to control pests in the field, in stored grain products (Giunti et al. 2018), and in urban areas (Britch et al. 2018). Pyrethrins have been used as competent pesticides with less well-known insecticide resistance traits in mosquitoes and other human pests while being safer in indoor domestic settings (Cuervo-Parra et al. 2016, Smith et al. 2016). Although considered an uncommon pest management technology, benefits to public health and future broader applications are undeniable as VOCs have proven to control insect vectors and reduce pathogen transmission while being a cost-effective alternative (Giles et al. 2011, Pokhrel et al. 2018). Incorporating VOCs into an IPM plan can reduce nontarget effects of classic pest control strategies, while providing enhanced suppression of pests through multiple modes of action.

## Team 4 Stance: Chemical Control Is the Best Technology to Control Undesirable Insect Pests in Urban and Agricultural SettingsTeam members: Jared E. Dyer, Na Xie, Jason Bielski, John SchepisProfessor Advisor: Dr. Doug Pfeiffer

When faced with an outbreak of agricultural or urban pests, management practitioners have historically relied on chemical controls for a simple reason—they work. Chemical controls provide consistent and reliable management of insect pests which has greatly improved the standard of living for billions of people and conferred great benefits to society (Copper and Dobson 2007). The use of chemical insecticides has allowed growers in many regions to maintain a profitable yield in lieu of significant decreases in crop losses due to pests (Oerke 2005). Simultaneously, the application of chemical insecticides has extensively improved the urban quality of life by keeping the public from infestation by annoying and deadly pests like cockroaches, bedbugs, and mosquitoes. The efficacy of pesticides has been well established through decades of research and will continue to improve with advancements in technology.

In the near future, agriculture may look more like a science fiction movie with the incorporation of drones, machine-based learning, and the Internet of Things (IoT). Precision agriculture is an interdisciplinary approach to pest management that attempts to encourage sustainable agriculture and data-driven decision-making to find long-term technology-based solutions to pest management (Iost Filho et al. 2020). It will be essential to mitigate the deleterious effects of chemical controls while also scaling up current agricultural production to meet the demands of a growing global population (Dara 2019). By using precision agriculture systems, or site-specific crop management, growers can identify the areas of a field that require pest management practices, nutrients, or rate overall crop health (Wang et al. 2019a, 2019b). Moreover, by identifying the intra-field variability, growers can reduce the overall chemical input required in the system (Sudbrink et al. 2015). Deployment of chemical controls through more precise means will thus allow for control of insect pests while also decreasing risks to external systems.

Advances in administering chemical control are not limited to precision agriculture and technology. Baits have been historically used in agricultural and especially urban settings showing effective control in versatile situations (Latchininsky and van Dyke 2006, Davari 2018). They can be applied more precisely compared to spray applications and the formulations can be tailored to be species-specific, preventing nontarget mortality (Buczkowski et al. 2014a, Davari et al. 2018). This can be done by mimicking the insect’s natural food with technologies such as hydrogels that make the bait appear like nectar (Buczkowski et al. 2014a). Direct interaction with these baits is not even necessary as social and semi-social insects will spread the bait horizontally to other members of the species (Buczkowski et al. 2008). Bait formulations can increase toxicity, control, and safety, and are another tool in the arsenal of chemical control.

Neonicotinoids, as an important component of chemical control, are one of the most popular classes of insecticides applied in pest management programs due to their high effectiveness and selectivity for insect pests, posing lower threats on nontarget organisms especially mammals because of their lower sensitivity to mammalian nicotinic acetylcholine receptors (Buczkowski et al. 2014b). Along with the development of advanced techniques like precision agriculture (Iost Filho et al. 2020), newer formulations (Singh et al. 2021), and hydrogel baits (Buczkowski et al. 2014a, 2014b), neonicotinoids are not only capable of exerting functions controlling urban and agricultural pests but decreasing the adverse effects on the environment and nontarget species to a greater extent. While historical abuses of synthetic pesticides have been a legitimate source of much controversy, these newer technologies and increasingly strict regulatory standards will ensure that chemical controls can meet the demands of a growing world while also mitigating adverse risks to the environment and public health.

## Compared to Other Solutions, Like Plant-Based Diets, Insect Farming Is the Best Method to Address Rising Human Global Food and Nutrient Supply Demands

### Unbiased Introduction by Victoria Pickens

Despite their consumption by humans worldwide for thousands of years, insects have gained popularity in the past decade as the “new” way to tackle world hunger and nutrient supply demands. Today’s food systems are unable to sustain our current population with safe and nutritious food (Hendriks et al. 2023), and the combination of climate change, environmental concerns, and the rapidly increasing human population further threaten the situation. One in 10 people around the world suffer from malnutrition (von Braun et al. 2023), and protein production will need to double to meet population estimates for 2050 (Steinfeld et al. 2006, Pelletier and Tyedmers 2010). Even by 2030, food demands are projected to increase 50% (Gahukar 2016). Consequently, global leaders and scientists are scurrying to find innovative solutions to tackle the human hunger crisis.

The human consumption of insects and related arthropods, or entomophagy, has been practiced throughout human history and remains popular in many areas of the modern world. As of 2013, the Food and Agriculture Organization of the United Nations estimated that at least 2 billion people consume insects on a regular basis (van Huis et al. 2013). Insects are being reevaluated as optimal sources for meeting global food and nutrient supply demands for a number of reasons. Compared to livestock and plant-based diets, insects often have higher protein contents, and are also efficient producers of other important nutrients and bioactives (Sun-Waterhouse et al. 2016). Lower usage of resources like water and land, in addition to lower environmental impacts and more rapid proliferation than livestock production, also make edible insects an ideal candidate. Insect farming, gathering, and/or processing is additionally a feasible option for nontraditional workers as it requires little infrastructure and is less labor intensive than most other food production systems (Dunkle and Payne 2016).

However, the question remains whether or not this is the best solution for tackling malnutrition. Unlike most other options, western culture has developed an aversion to insect consumption. Efforts would have to be made by marketers, policymakers, and industry to change perceptions of consuming insects due to psychological or cultural barriers by promoting awareness of insects and insect-based foods (Batat and Peter 2020). Instead, consumers may more readily embrace other food alternatives for a sustainable diet that are readily available, such as plant-based diets that substitute plants for animal-based proteins. Food safety is also a larger concern for insect production, as currently there are unknowns about potential risks of chemical and microbial contaminants associated with rearing, consumption, or their use as food ingredients (Imathiu 2020). With so many unknowns, it is difficult to establish proper methods for ensuring food safety of these products. In Western cultures such as the European Union, United States, and Canada, edible insects and insect products are regulated as a “novel food”, whereas other countries where insects are more habitually harvested and consumed, like China and Mexico, do not have a specific regulatory framework. Currently, there is no consistent regulation internationally, limiting global trade of this commodity (Lähteenmäki-Uutela et al. 2021). Instead of attempting to change diets and cultural opinions, other methods instead argue for tackling current food system problems like maximizing crop or animal production, using other food alternatives, improving distribution efforts, or reducing food waste. While entomophagy certainly isn’t a new concept, it is a potential solution to rising malnutrition around the globe. However, there are other viable solutions as well which may be superior. Considering the rapid expansion of the human population and our limited resources, it is vital to identify and pursue the optimal methods for rising human food and nutrient supply demands.

## Team 5 Stance: Compared to Other Solutions, Like Plant-Based Diets, Insect Farming Is the Best Method to Address Rising Human Global Food and Nutrient Supply DemandsTeam Members: Kayleigh C. Hauri, John J. Ternest, Jacob Pecenka, Scott W. Gula, Natalie ConstancioFaculty Advisor: Dr. Zsofia Szendrei

Global malnutrition and hunger are expected to worsen with population growth, requiring major changes to food production systems (van Huis et al. 2013). Insect farming is a promising option to address increased food demand due to its environmental sustainability, high economic return, and potential for widespread adoption.

In the developing world, where most human population growth is expected, the increase in livestock products is estimated to increase by 70% in the coming decades (van Huis and Oonincx 2017, Chia et al. 2019). While plant-based alternatives are being developed, a fully plant-based diet does not provide all necessary nutrients for development, especially in developing countries (Ingenbleek and McCully 2012). A more sustainable alternative to a plant-based diet is an insectivore diet. Mealworms require only 1/1,000 the water/kg as cattle do, representing an enormous opportunity to improve the conditions of the nearly 2 billion people worldwide with insufficient water supplies (Naseem et al. 2020). Several insects, as both feed ingredients or for human consumption, were found to be more efficient at converting feed and had a higher nitrogen-efficiency than conventional livestock (Oonincx et al. 2015). In some cases, pest insects can be successfully harvested and exploited for human consumption while simultaneously reducing chemical inputs and pest management costs (Cerritos Flores et al. 2015).

Insect farming and harvesting require little space, making it accessible to landless people (Cerritos Flores et al. 2015, Naseem et al. 2020). Additionally, insect farming requires low labor inputs (Hanboonsong et al. 2013), meaning insect farming can be done by groups that are traditionally excluded from farming, such as women, the elderly, or people with disabilities. Many insects can be farmed with almost no additional crop production (Gahukar 2016). For example, the black soldier fly can eat agricultural byproducts and portions of crops that are inedible to humans. Careful choice of insect species could reduce food waste and provide a high-value protein and calorie source without putting additional land into production. Additionally, the overall production costs of insect farming are lower than livestock production (Gahukar 2016). Farmers in Thailand make an average annual income of US$5000/yr pursuing cricket farming compared to US$2200/yr for crop cultivation (Gahukar 2016). Unsurprisingly, in Thailand cricket farming has shifted from secondary to primary income source for many farmers and is now a multi-million dollar industry that provides jobs for tens of thousands of people (Hanboonsong et al. 2013).

Entomophagy, while a common practice worldwide, is not widely practiced in Western countries, largely due to cultural aversion. Many arguments promoting the adoption of entomophagy in Western cultures have attempted to appeal to people’s rationale, comparing the sustainability of insect-based diet to animal or plant-based food, and citing global malnutrition (Shockley and Dossey 2014). However, these arguments create the “insectivore’s dilemma”: we are told that entomophagy should be widely adopted without being given appetizing options to choose from (Deroy et al. 2015). Fortunately, these barriers can be overcome through reeducation and a change in cultural appreciation (Looy and Wood 2006, Yen 2009). Surveys of university students pre- and postexposure to insects as food showed that after an “insect experience” there was an increased positive reaction to insects as food (Looy and Wood 2006). Additionally, arguing that entomophagy is unrealistic ignores the current use of over 1900 species as a portion of traditional diets by over 2 billion people (van Huis et al. 2013). Food safety concerns exist for all commodities, but edible insects are considered to pose a low risk of disease or pathogen transmission using existing techniques of sanitation and food preparation (Liceaga 2019, Doi et al. 2021).

## Team 6 Stance: Compared to Other Solutions, Like Plant-Based Diets, Insect Farming Is Not the Best Method to Address Rising Human Global Food and Nutrient Supply DemandsTeam Members: Emily Rampone, Mario Luppino, Dowen Jocson, Stephen Onayemi, Emily RendlemanFaculty Advisor: Dr. David Crowder

One of the key challenges for agriculture is to promote global food security while limiting negative externalities such as emissions. However, agricultural ecosystems currently account for 34% of global carbon emissions, and food security remains a worldwide problem, especially in the face of climate change. While insect farming has been proposed as one solution to address these problems, there are better options to reduce agricultural emissions and increase food security. A holistic approach that alters diets to reduce meat consumption, invests in soil health measures, and curtails food waste that can meet global food needs and reduce emissions (Muller et al. 2017).

When considering how agriculture can continue to provide food security, researchers often concentrate on developing countries. This minimizes the nutritional deficiencies in Western diets and perpetuates colonial ideals of white saviorism. Western diets over-consume protein and lack plant nutrients (Delimaris 2013). Consumers seeking sustainable, environmentally conscious diets are often choosing plant-based diets (Vinnari and Vinnari 2014), and Westerners are more accepting of plant-based diets than entomophagy due to long-standing cultural biases (Defoliart 1999). We should increase education and promotion of plant-based diets in Western societies with focus on reducing emissions and increasing food security.

Soil management affects global emissions. By regeneratively managing farmland, we can produce sustainable protein while sequestering carbon. Destruction of farmland soils has increased CO_2_ emissions over the last century, currently accounting for ~10% of global emissions (Amundson et al. 2015). Transitioning agricultural systems to organic, high-protein crops is more achievable and economical than creating insect farming industries (Muller et al. 2017). Investments in sustainable agriculture can reinvigorate soil health, diversify farmer income streams, and reduce pesticide and water use (LaCanne and Lundgren 2018).

There are many reasons not to pursue insect farming specifically. First, managed species serve as reservoirs for pathogens that can be transmitted to wild populations (Colla et al. 2006). Transmission is often high near insect rearing facilities, suggesting that increased numbers of facilities would increase the risk of spillover (Otterstatter and Thomson 2008). Spillover not only harms wild insects but overall ecosystem functioning (Basset and Lamarre 2019). Insect farming can also be a source of human illness. Mealworms require treatment to reduce bacterial loads, accounting for up to 10% of the insect’s biomass, and have been found at levels higher than those recommended in minced meat (Klunder et al. 2012). Additionally, insects carry molds that cause allergic reactions (Imathiu 2020). Pathogens like rat tapeworm can be transmitted to humans and livestock, causing intestinal distress (Grau et al. 2017).

Most mass-produced insects are also raised in heated rooms and then processed. This uses tremendous energy, potentially negating emission reductions from choosing an alternative protein source (Oonincx et al. 2015). Notably, water use in mealworm production is comparable to chicken production, while energy use is comparable to beef production (Grau et al. 2017). Whereas curtailing food waste can ensure we are maximizing our existing resources and production streams. The feed used to produce the 20% of meat that is wasted could be used to feed 235 million people (Davis and D’Odorico 2015).

In conclusion, to address climate change and global hunger, we should bolster established technology and supply chains to transition rather than invest in new ones. Moreover, efforts to address hunger or climate change in the developing world should be led by residents of those countries. The AFSA (2017) highlights the importance of using agroecological practices and moving away from subsidized food imports, which destabilize local agricultural economies. Western society should refocus on these sustainable transitions to fix our ineffective infrastructures rather than expect the rest of the world to compensate.
